# Spatially-explicit valuation of coastal wetlands for cyclone mitigation in Australia and China

**DOI:** 10.1038/s41598-018-21217-z

**Published:** 2018-02-14

**Authors:** Xiaoguang Ouyang, Shing Yip Lee, Rod M. Connolly, Martin J. Kainz

**Affiliations:** 1 Australian Rivers Institute, School of Environment and Science, Griffith University Gold Coast campus, QLD, Queensland, 4222 Australia; 2 Simon F.S. Li Marine Science Laboratory, School of Life Sciences, and Earth System Science Programme, The Chinese University of Hong Kong, Shatin, Hong Kong SAR China; 3 WasserCluster Lunz–Inter-university Centre for Aquatic Ecosystem Research, A-3293 Lunz am See, Austria; 4 Sichuan Zhonghuanlixin Environmental Protection Consulting Co., Ltd., Sichuan Province, Bazhong, 636000 P.R. China

## Abstract

Coastal wetlands are increasingly recognised for their pivotal role in mitigating the growing threats from cyclones (including hurricanes) in a changing climate. There is, however, insufficient information about the economic value of coastal wetlands for cyclone mitigation, particularly at regional scales. Analysis of data from 1990–2012 shows that the variation of cyclone frequencies is related to EI Niño strength in the Pacific Ocean adjacent to Australia, but not China. Among the cyclones hitting the two countries, there are significant relationships between the ratio of total economic damage to gross domestic production (TD/GDP) and wetland area within cyclone swaths in Australia, and wetland area plus minimum cyclone pressure despite a weak relationship in China. The TD/GDP ratio is significantly higher in China than in Australia. Despite their extensive and growing occurrence, seawalls in China appear not to play a critical role in cyclone mitigation, and cannot replace coastal wetlands, which provide other efficient ecosystem services. The economic values of coastal wetlands in Australia and China are respectively estimated at US$52.88 billion and 198.67 billion yr^−1^ for cyclone mitigation, albeit with large within-country geographic variation. This study highlights the urgency to integrate this value into existing valuations of coastal wetlands.

## Introduction

Coastal wetlands play a pivotal environmental and economic role in protecting coastlines from cyclones, mitigating climate change through carbon sequestration and storage, serving a nursery function for fisheries or recreation function for humans, and mediating the effects of floods or enhancing water quality^1–5^. Protection from cyclones is paramount due to the overwhelming trend of coastal urbanisation^6,7^. Coastal wetlands attenuate the potential catastrophic damage of cyclones, relying on their ability to dissipate storm energy^8–10^. The natural shield provided by wetlands outweighs engineering measures, such as seawalls and bulkheads^11,12^, which have a defined lifespan, require continual maintenance, and could not replace the fishing, recreational and other roles of coastal wetlands. Such structures can also be costly to build, and exacerbate coastal erosion or cause knock-on effects in neighbouring regions^13,14^. Despite these shortcomings, hard-engineering options such as seawalls are often the preferred options, resulting in extensive irreversible modification of the coastline, e.g. the ‘New Great Wall’ of China^15^. Nonetheless, so far, no studies have quantitatively determined the advantage of coastal wetlands over seawalls from the perspective of ecosystem services.

Valuations of wetland protection from coastal natural disasters (including cyclones) generally fall into two classes: ecological or functional valuation and economic or monetary valuation. Increasing academic attention has been paid to the role of wetlands in shielding coastal regions from cyclones since the 2004 Indian Ocean tsunami. Studies have assessed the impact of coastal natural disasters based on anecdotal evidence^16^, post-hoc observations^17^, remote sensing, and modelling^18^. The validity of such studies is limited by the ability to constrain confounding factors. Thus, there is on-going debate over the conclusions of such studies. Lee, *et al*.^10^ stated that remote sensing may be ineffective in detecting cryptic degradation, resulting in fragmented mangroves, which may not provide the protection that intact mangroves can. Pinsky, *et al*.^19^ stressed the controversial function of coastal wetlands for storm protection due to the variation of geomorphic, ecological and hydrodynamic factors that determine wave attenuation among locations and times. Further, most studies used whole countries as units of observation, and may thus miss important spatial variations within a country. It is paramount to conduct studies focusing on a spatially explicit evaluation of the role of wetlands in protection from cyclones at both between-country and within-country scales.

On the Western Pacific rim, China and Australia are the largest countries in the northern and southern hemispheres, respectively; both with long coastlines. Coastal regions of both countries are subject to intense cyclone activities^20^ with potentially devastating ecological and economic effects. However, these two countries differ in their natural assets potentially protecting them from cyclones, with large differences in geographic extent and distribution of natural wetlands, ocean meteorological conditions and mitigation options. For example, China has established seawalls along the coastline, reaching 11,000 km in extent by 2010^15^, as an alternative to natural wetlands for protecting the coastal regions from cyclones. Such infrastructure is uncommon in Australia, except some urban areas. On the other hand, Australia regularly suffers from the significant direct impact of EI Niño Southern Oscillation, e.g. sustained drought^21^. Further, based on the recent global maps of mangroves^22^ and saltmarshes^23^, in coastal wetlands of China, the distribution of mangroves is constrained to subtropical regions, while the distribution of saltmarshes covers almost the whole coastline. In contrast, mangroves and saltmarshes occur almost concomitantly along the coastline in Australia.

Costanza, *et al*.^8^ pioneered the study of valuing wetlands in mitigating 34 cyclones in the USA over the period 1980–2004. However, their study was restricted to the land use map of one year (i.e. 2000) to extract decadal wetland area. Their study also used only wind speed as an indicator of cyclone intensity, while barometric pressure predicts damage more accurately than maximum sustainable wind speed^24^. Barbier^25^ recommended the method of estimating protective services of wetlands in terms of the reduction in the expected damages or deaths inflicted on coastal communities.

The objectives of this study are to: (a) analyse different characteristics (including frequencies and minimum central pressure) of cyclones occurring in oceans around Australia and China during 1990–2012; (b) analyses of the relationship between TD/GDP and wetland area in cyclone swaths, wind speed, and the duration and minimum central pressure of cyclones for both countries separately; (c) assess the value of Australian and Chinese wetlands in protecting the countries from cyclones over the period 1990–2012; (d) assess the influence of El Niño on cyclone activities in both countries; (e) the effect of seawalls on TD from cyclones in China, in combination with a cost-benefit analysis to compare seawalls and coastal wetlands in their efficacy for cyclone mitigation. In this study, we followed the avenue of Costanza, *et al*.^8^ who estimated the value of wetlands in mitigating hurricanes hitting the USA from the relationship between the ratio of total economic damage to gross domestic production (TD/GDP) and influential variables. However, we refined their method by including the minimum central pressure as an indicator of cyclone intensity (for both China and Australia in the initial regression model) rather than maximum wind speed alone. We used time-series land-use maps in our analysis and also assessed the effect of El Niño on cyclone occurrence, as well as the impact of seawalls on cyclone mitigation in China.

## Results

**Figure 1:**
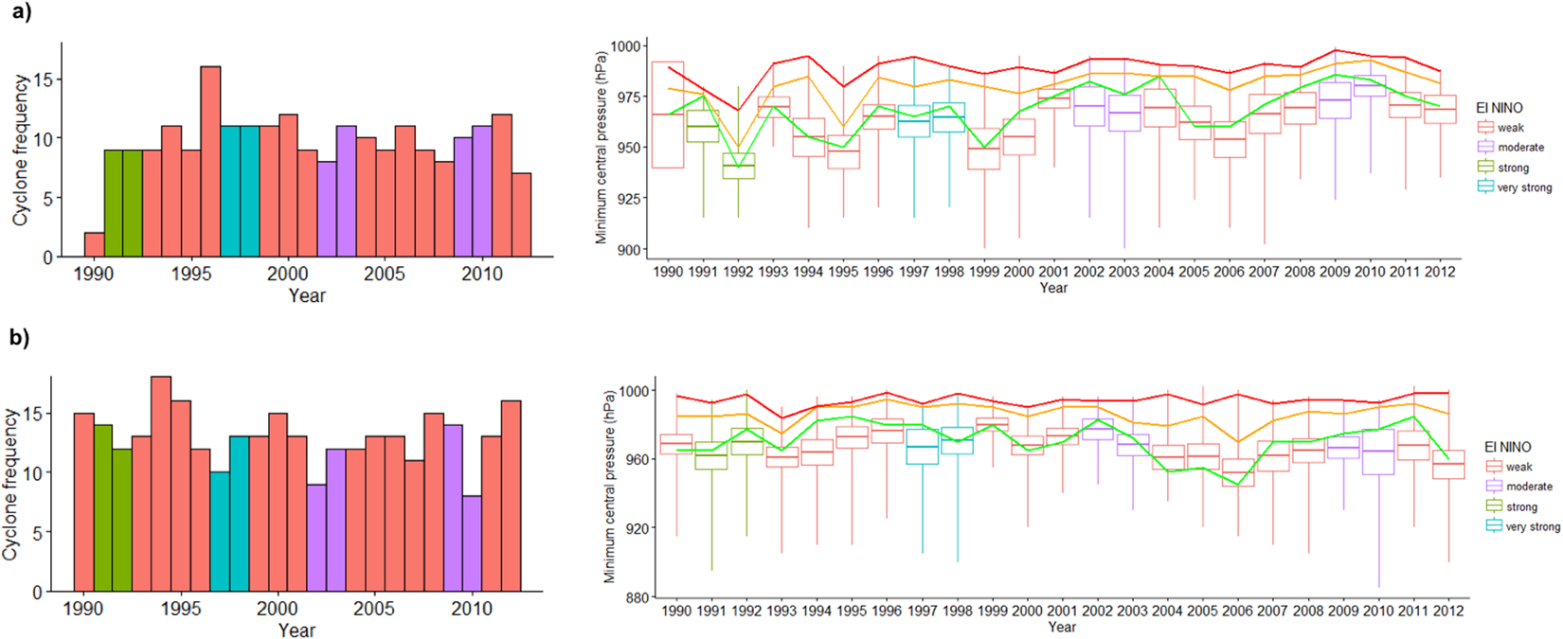
Variation of cyclone frequencies and minimum central pressure with El Niño events in Australia (**a**) and China (**b**) during 1990–2012. Boxplots show the variation of minimum central pressure during the period. Trend lines with green, orange and red colours were added for the median, 75% and 95% quantiles of minimum central pressure.

We assessed the variation of cyclone frequencies and intensities along with the occurrence of El Niño events. Of the 531 cyclones in Australia and China during 1990–2012, no clear temporal trend in cyclone frequencies is found in the oceans around either Australia or China (Fig. 1). Nevertheless, during the very strong El Niño events in 1997–1998, cyclones were frequent in the oceans around Australia. However, no similar temporal trend is found in the ocean around China. Similarly, no clear temporal trend could be observed for cyclone intensities (i.e. minimum central pressure) in the oceans around either Australia or China during 1990–2012, even combined with El Niño intensities (Fig. 1).

**Figure 2:**
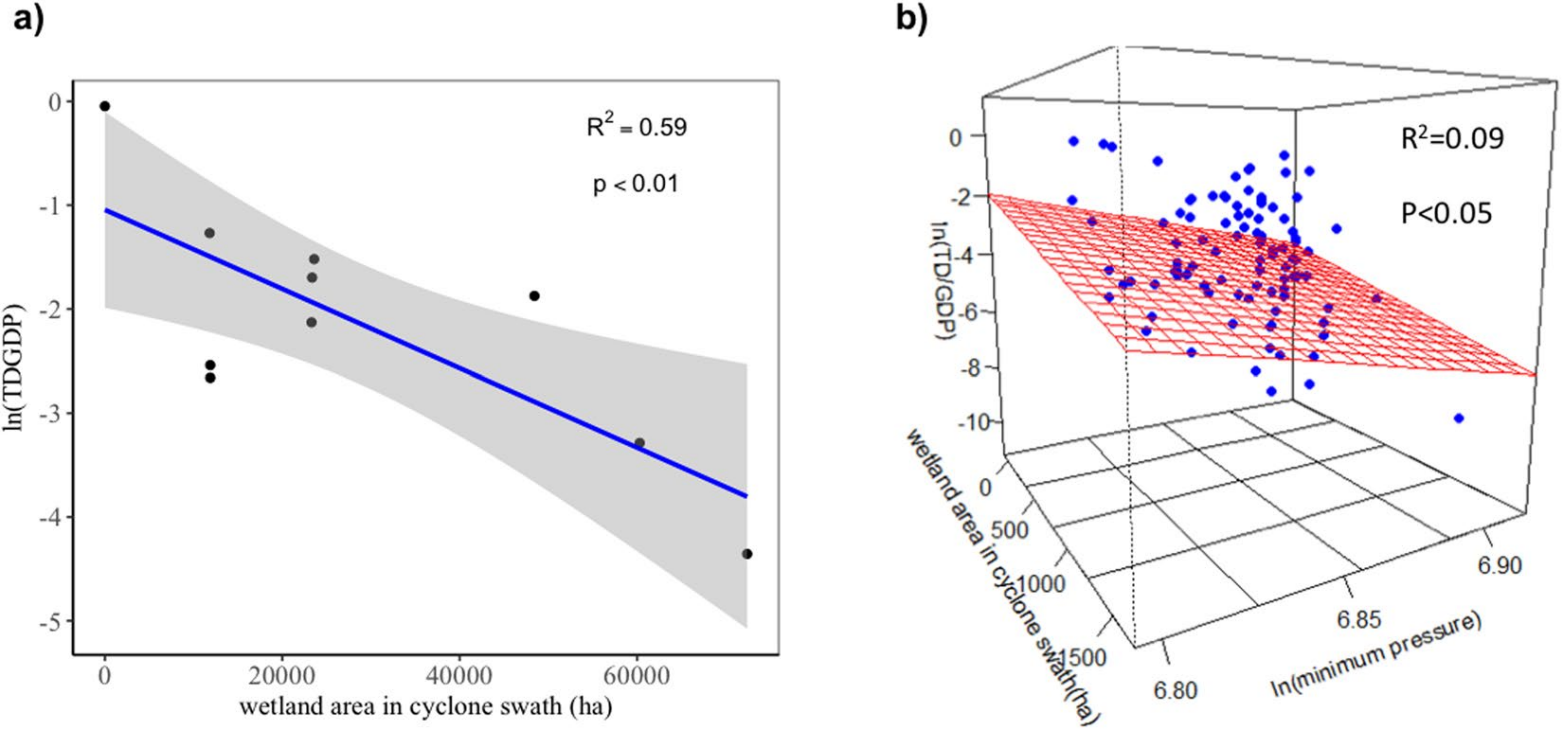
The relationship between TD/GDP and wetland area in cyclone swaths and/or the minimum central pressure of cyclones in Australia (**a**) and China (**b**). The regression equation in (**a**) $ln\frac{TD}{GDP}=-1.044-3.812\,\times$ 10^−5^ × weltland area in cyclone swath. The shaded area is 95% confidence intervals. The regression equation in (**b**) $\,ln\frac{TD}{GDP}=123.7-1.475\times {10}^{-3}$ × weltland area in cyclone swath −18.52 × ln (minimum pressure).

We conducted regression analysis to examine the relationship between TD/GDP and wetland area, wind speed, the duration and minimum central pressure of cyclones for both countries, but non-significant independent variables were excluded. Finally, a significant negative linear relationship exists between ln (TD/GDP) and wetland area along the cyclone swath in Australia (R^2^ = 0.59, p < 0.01, supplementary text, Table S1 and Fig. 2a). Likewise, there is a significant negative relationship between ln (TD/GDP) and a combination of ln (minimum pressure) and wetland area in cyclone swath) in China (R^2^ = 0.09, p < 0.05, Akaike’s Information Criterion (AIC) = 408.3, supplementary text, Table S1 and Fig. 2b). The variance explained by ln (minimum pressure) and wetland area in cyclone swaths account for 51.4% and 48.6%, respectively. The other and only regression model valid for data in China is the relationship between ln (TD/GDP) and wetland area in cyclone swaths, the AIC of which is 410.3, higher than the previous model, and was discarded.

**Figure 3:**
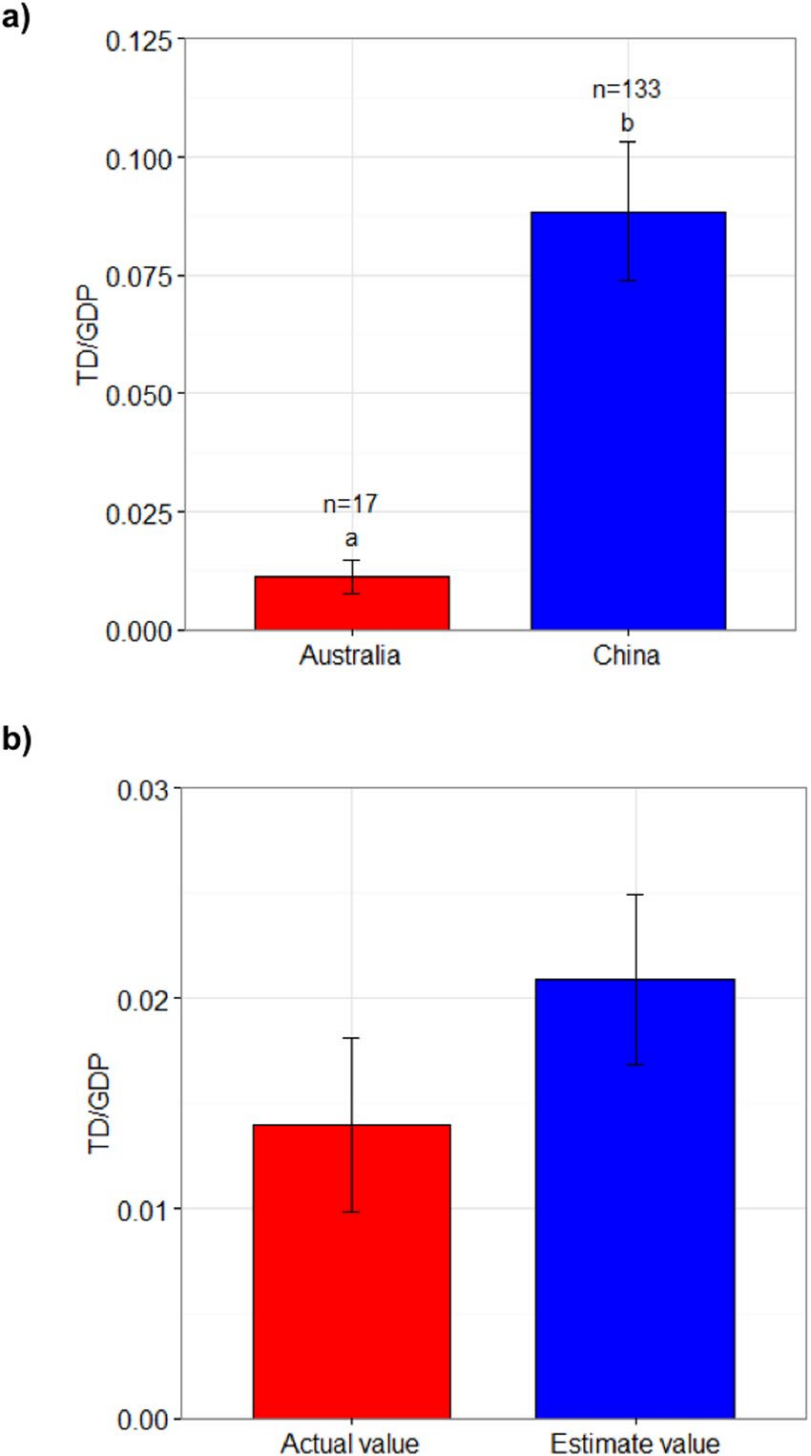
Comparisons of TD/GDP in relation to the cyclones during 1990–2012 in China and Australia (**a**), and actual and estimated TD/GDP in relation to cyclones after 2010 in China (**b**). Bars labelled with different letters are significantly different from each other (Mann-Whitney test). Data are mean ± standard error.

We evaluated the effect of seawalls on the TD from cyclones in China by comparing the actual and estimated TD/GDP derived from the regression model after 2010, when seawalls were established along the China’s coastline. For the two countries, TD/GDP for the cyclones was significantly higher in China (0.0884 ± 0.0147) than in Australia (0.0113 ± 0.0036) (Mann-Whitney test, p < 0.01) (Fig. 3a). Based on the above linear relationship, after 2010, the estimated value of TD/GDP (0.0209 ± 0.004) is not significantly higher than the actual value (0.0139 ± 0.004) in China (paired t-test, p > 0.05; Fig. 3b).

We derived the annual marginal value (MV) of coastal wetlands in both countries from the final regression models. MV is the value of unit wetland area in protecting wetlands from cyclone attack. We show the estimated MV in an average hurricane swath (MV_sw_), the total value (TV_s_) (supplementary text) for all the coastal wetlands and the unit average annual value of coastal wetlands for each state (or province, municipality) (Table 1). Annual expected MV per average swath is highest in the Northern Territory ($49,240 ha^−1^ yr^−1^), intermediate in Western Australia ($44,502 ha^−1^ yr^−1^), and lowest in Queensland ($39,067 ha^−1^ yr^−1^), whereas the total value of wetland protection ranks in the reversal order, i.e., $15.6 billion y^−1^, $16.1 billion y^−1^ and $21.2 billion y^−1^. The annual expected MV per average swath (total value of wetland protection) also shows large variability in China, ranging from the highest value $585,022 ha^−1^ yr^−1^ ($50.6 billion y^−1^) in Zhejiang, to as low as $3,647 ha^−1^ yr^−1^ ($118 million y^−1^) in Shanghai.

**Table 1:** Annual expected MV per average cyclone swath and total value of wetland protection in relation to cyclones.

State	wetlands within 100 km of coast by state W_s_ (ha)	Wetland area in average swath W_sw_ (ha)	GDP in average swath (million $ yr^−1^)	Probability of state being hit by cyclones of a category by cyclone category (%)					Annual expected MV per average swath MV_sw_ ($ ha^−1^ yr^−1^)		Total value of wetland protection TV_s_ (million $ yr^−1^)
				1	2	3	4	5			
**(1) Australia**											
WA	361744	5281	10364	4.35	0.00	8.70	8.70	17.39	44502		16098
NT	316526	33542	33678	8.70	4.35	8.70	0.00	17.39	49240		15586
QLD	542616	8385	13167	4.35	0.00	4.35	4.35	17.39	39067		21198
**(2) China**											
**Province/Municipality**	**wetlands within 100 km of coast by state W**_**s**_ **(ha)**	**Wetland area in average swath W**_**sw**_ **(ha)**	**Minimum central pressure of cyclones**	**GDP in average swath (million $ yr** ^**−1**^ **)**	**Probability of state being hit by cyclones of a category by cyclone category (%)**					**Annual expected MV per average swath MV**_**sw**_ **($ ha** ^**−1**^ **yr** ^**−1**^ **)**	**Total value of wetland protection TV**_**s**_ **(million $ yr** ^**−1**^ **)**
					1	2	3	4	5		
Shanghai	32400	123	965	1212	8.70	0.00	0.00	0.00	0.00	3647	118
Hong Kong	10800	297	956	4187	17.39	4.35	0.00	0.00	0.00	28979	313
Guangxi	108100	478	958	8892	69.57	30.43	4.35	0.00	0.00	218785	23651
Guangdong	216200	436	952	9525	73.91	52.17	13.04	0.00	0.00	371403	80297
Zhejiang	86500	80	945	15390	34.78	17.39	17.39	0.00	0.00	585022	50593
Jiangsu	64900	384	931	4423	0.00	4.35	13.04	0.00	0.00	35205	2283
Hainan	10800	135	956	4987	30.43	17.39	4.35	0.00	0.00	105358	1139
Fujian	75700	79	940	7052	56.52	30.43	34.78	4.35	0.00	532193	40271

Note: WA denotes Western Australia, NT denotes Northern Territory and QLD denotes Queensland.

## Discussion

The variation of cyclone frequencies and minimum central pressure reflects the patchiness in cyclone occurrence and intensities. The frequent occurrence of cyclones in the oceans around Australia in 1997–1998 reflects the accumulated energy in the Southern West Pacific Ocean during the very strong EI Niño events. The lack of similar trends for minimum central pressure in the oceans around either Australia or China during 1990–2012 is probably related to the interannual variability of sea surface temperatures (SST)^26^.

Our study sheds light on the effect of wetlands in reducing the economic damage by cyclones for both countries. The regression models show that TD/GDP decreases with increasing wetland area in Australia, and with increasing wetland area and minimum central pressure in China. This negative effect of wetland area on TD/GDP is in accordance with that of Costanza, *et al*.^8^, although the regression models derived in the two studies are different. The study by Costanza, *et al*.^8^ assessed the annual economic value of coastal wetlands in shielding hurricanes, taking into account maximum sustainable wind speed, while the current study uses the minimum pressure as an estimate of cyclone intensity. This study further demonstrates that wetlands, especially coastal wetlands, are crucial in attenuating the impact of cyclones^27,28^. The R^2^ values of the results for both countries (Australia: 0.59; China: 0.09) are lower than that of the analysis on USA (0.6). This suggests that (1) there may be other variables explaining the variation of TD/GDP in Australia and China, in comparison with USA; or (2) the uncertainty in cyclone damage measurement may explain part of the difference, in particular for China, which has limited avenues to precisely quantify cyclone damage in the 1990s.

As shown in Ma, *et al*.^15^, seawalls have been established extensively along the coastline of China since 2010, while wetlands reclaimed for seawall construction had the fastest areal increases during 1991–2010. This means that seawalls have replaced natural wetlands and have been the main mitigation measure for cyclones after 2010. Since seawalls were being continuously built and were not complete until 2010, the estimated value of TD/GDP is calculated without the effect of seawalls that could not be included as an independent variable in the regression model, while the actual value is based on relatively complete seawalls. Nevertheless, no significant difference in TD/GDP is found between the estimated and actual values, suggesting that seawalls may not provide significant protection of the coastlines. In addition to indicating the insignificant effect of seawalls for cyclone mitigation, we have collected and analyzed benefit/cost ratio data to show other ecosystem services provided by ‘green infrastructure’ (i.e. wetlands) but not provided by ‘grey infrastructure’ (including seawalls). The benefit/cost ratios were calculated as the ratio of the benefits of ecosystem services and the costs arising from service provision, e.g. operation and management. Wetlands provide other important ecosystem services, including wastewater treatment, recreation, carbon sequestration, flood control and fisheries (Table 2). The cost-benefit analysis reflects that wetlands have a high benefit/cost ratio (>5) for wastewater treatment and moderate benefit/cost ratio (>1) for recreation and carbon sequestration. No cost-benefit relationships were found for flood control and fisheries but both have high economic values ($971–35,034 ha^−1^ yr^−1^ for flood control and $2,805–17,090 ha^−1^ yr^−1^ for fisheries).

**Table 2:** The cost-benefit/benefit analysis of important ecosystem services provided by wetlands in addition to cyclone mitigation (values were sourced from the stated references).

Ecosystem services	Indices	Indices value	References
Wastewater treatment	Benefit/cost	5.2–6.5 (dollar-based) 8.8–22.1 (energy-based)	Ko, *et al*.^48^
Recreation and carbon sequestration	Benefit/cost	1.01–1.03	Ghermandi and Fichtman^49^
Flood control	Benefit	$971–35034 ha^−1^ yr^−1^	Woodward and Wui^50^; Camacho-Valdez, *et al*.^51^
Fisheries	Benefit	$2805–17090.1 ha^−1^ yr^-1^	Woodward and Wui^50^; Mukherjee, *et al*.^52^

This result of TD/GDP for the cyclones in Australia and China is in agreement with the present (1981–2000) and future (2081–2010) baseline estimate of TD from tropical cyclones that TD in East Asia was significantly higher than that in Oceania. In particular, TD in China was one order of magnitude higher than that in Australia for the future baseline estimate^24^.

The result of wetland value for cyclone mitigation in this study is comparable to previous studies evaluating the protective role of coastal wetlands for hurricane mitigation. Costanza, *et al*.^8^ estimated MV_S_ between $ 256 ha^−1^ yr^−1^ and $51,000 ha^−1^ yr^−1^ for 19 states in the USA and $3,228 ha^−1^ yr^−1^ for the entire USA (median value). The valuation of wetlands for cyclone mitigation shows that the values (from $39,067 ha^−1^ yr^−1^ to $49,240 ha^−1^ yr^−1^) for the states/territory in Australia just fit in the range of some states in USA, while the values (from $3,647 ha^−1^ yr^−1^ to $58,502, 2 ha^−1^ yr^−1^) for the provinces/municipalities in China are generally at the higher end of the range. The overall values of coastal wetlands in Australia and China are $52,882 million and $198,666 million yr^−1^, respectively. The values for Australia are higher than the high and middle percentile ($12,765 million yr^−1^ and $23,162 million yr^−1^), but lower than the low percentile of values ($87,330 million yr^−1^) in USA, while the values for China are higher than the low percentile of values in USA. The difference may partly lie in the limited available data for estimation in USA and Australia since the frequency of cyclone incidence is a positive factor in determining the values. The USA data only include major cyclones (i.e. hurricanes), while cyclone damage is poorly reported in Australia and largely unknown. On the other hand, the weak regression model for China can result in large uncertainties in the value of wetlands, and our comparison with the wetland value of Australia and USA. Barbier^29^ used the Expected Damage Function to estimate the protective value of mangroves in Thailand against coastal natural disasters, and derived a value of $5,850 ha^−1^ yr^−1^, which is lower than the estimated range ($28,979–532,193 ha^−1^ yr^−1^) of adjacent regions in China, including the municipality of Hong Kong, and the provinces of Guangxi, Guangdong and Fujian. The difference may lie in different wetland area and GDP in cyclone swaths, as well as the different methods used to estimate the values.

**Figure 4:**
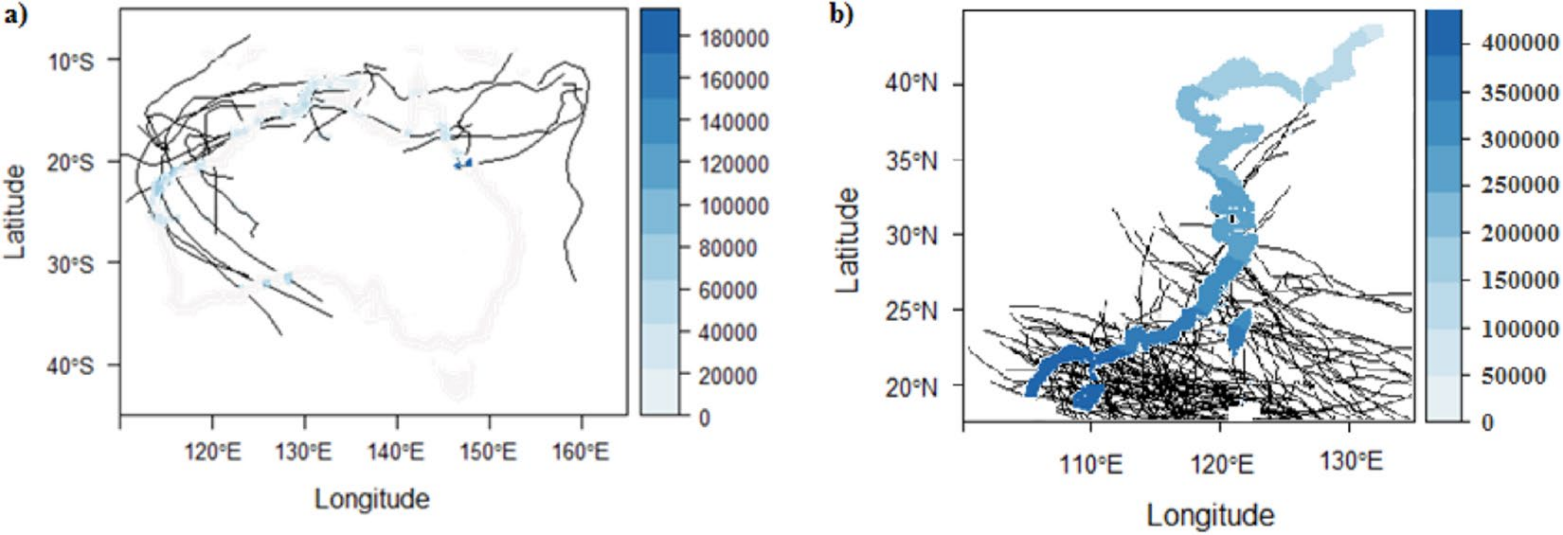
Annual expected MV for an average cyclone swath in the coastal regions in Australia (**a**) and China (**b**). The coastal regions are constrained within 100 km of the coastline. Cyclone tracks are the lines in black. The figure was generated by R programming language (R version 3.3.1, URL: https://www.R-project.org/).

The annual expected MV for an average cyclone swath in the coast regions varies widely between $0 and 2 × 10^5^ ha^−1^ yr^−1^ in Australia, and more widely between $0 and 4 × 10^5^ ha^−1^ yr^−1^ in China (Fig. 4). In Australia, the annual expected MV is higher in the northern than the southern coastline, while the reverse is true in China (Supplementary Material Table S2). Hurricane and cyclone activity generally occurs over the ocean in regions where SST exceeds 26 °C^30^. The northern coastline in Australia and southern coastline in China belong to tropical and/or sub- tropical regions, and SSTs are relatively high, compared to other coastlines in both countries. These sections of the coastlines of both countries suffer from more intensive cyclone disturbance than other coastal regions, resulting in their relatively high annual expected MVs.

The annual expected MV per average swath has both similarities and differences to previous studies on the protective value of coastal wetlands. The differences may be due to the difference in value of various wetland types in cyclone mitigation. Barbier, *et al*.^31^ reviewed the value of coral reefs, salt marshes and mangroves for cyclone protection, and found that the value ranged from $174 ha^−1^ yr^−1^ to $10,821 ha^−1^ yr^−1^. In addition, these marine habitats are effective in dealing with different disasters^18,20^. For example, mangroves are most effective in attenuating storm-induced waves less than 6 m in height^28^, and the occurrence of a belt of mangroves of 100 m can attenuate wave height remarkably (6 m in height)^32,33^, owing to their complex aerial root structure^34^. Further, there are other factors contributing to the variation of protective value with reference to coastal wetlands, such as distances from the coast. The protection value tends to decrease with increasing distance onto land.

There are uncertainties and limitations in our estimates. Firstly, globally, cyclone damage is not well measured in all countries^24^ and this also applies to the countries evaluated herein. There is no international consensus in terms of best practices for collecting these data, and thus there remains considerable variability in reporting them. In particular, there is a lack of reported cyclone damages that hit Australia, including (1) cyclones reported with unknown damages; (2) cyclones reported with minor damages and unable to be quantified; and (3) anonymous cyclones, damage data of which cannot be found. This results in the patchy data in the regression analysis and some influence on our valuation of wetlands for cyclone mitigation. In particular, the probabilities of cyclone occurrence at intermittent categories in some states are zero, and may generate uncertainties for the valuation of wetlands in Australia. From the perspective of natural disaster management, Australia needs to strengthen the estimation of cyclone damages to facilitate effective disaster compensation after cyclone attack. Secondly, the impact of climate and human forces on cyclone damage could not be accounted for in our regression model. Strong interannual variability in cyclone statistics and the possible influence of interannual variability associated with EI Niño events make it difficult to discern any temporal trend relative to background SST increases with correlational accuracy^26^. Consequently, any variability of SSTs associated with EI Niño events could not be explained in the model for Australia. On the other hand, precise geographic information about seawalls during the study period (1990–2012) could have further improved the model for China, but these data were unavailable. Finally, the weak regression model accounts for some uncertainties in the comparison of actual versus estimated TD/GDP, and the valuation of wetlands in China. Coastal wetlands have clear zonal compositions in different geographic regions of China, i.e. both mangroves and saltmarshes occur in subtropical regions but only saltmarshes in temperate and cold regions. Mangroves are trees while saltmarshes consist of grasses, sedges, bushes or other herbs. The physio-morphological difference of vegetation in the two types of coastal wetlands may explain part of the differences in the damage resulting from cyclones, but this is outside the scope of this study. Future studies are expected to improve the precision of wetland valuation if the area of different coastal wetland types can be quantified for each year of the study period.

## Methods

We followed the approach of Costanza, *et al*.^8^ who estimated the value of wetlands in mitigating hurricanes hitting the USA from the relationship between TD/GDP and influential variables.

### Data collection and collation

Data on the cyclones were assembled from pertinent web sites. First, we collected available data on the main cyclones (maximum sustained wind speed >120 km h^−1^) striking Australia (17 of the total of 231) and China (97 of the total of 300) since 1990. TD information for 17 cyclones in Australia and 97 cyclones in China that were regarded as ‘disasters’ was collected from www.emdat.be, which provides an objective basis for vulnerability assessment and rational decision-making in disaster situations, including disaster-related economic damage estimates. TD was transferred to US dollars of the base year (2011) based on implicit price deflator from World Bank. Based on established classification schemes (www.nhc.noaa.gov/climo), cyclones are classified as tropical depression, tropical storm and hurricanes (including major hurricanes), depending on the maximum sustained wind speeds. Where reported damages were remarkably different from this website, the cyclones were removed from our analysis, such as cyclone Yasi in 2011. Second, tracks of all cyclones hitting Australia and China from 1990–2012 were collected from www.bom.gov.au and relevant literature^35^. Night-time light imageries of Australia and China come from NOAA’s National Geophysical Data Centre. GDP was a good surrogate for economic activity and established infrastructure. GDP of both countries was collected from www.data.worldbank.org. Like TD, GDP was transferred to US dollars of the base year. We followed the method of Costanza, *et al*.^8^ to allocate GDP. A one-kilometre resolution GDP map was plotted by a linear allocation of the actual national GDP to the night time light intensity values^7^. Third, the 0.1° resolution land cover dataset of each year were collected from www.grid.unep.ch. TD and GDP estimated within each swath were utilized to entrain a ratio (TD/GDP), which represents the relative economic damage of each cyclone.

### Data analysis

The fluctuation of cyclone frequencies and intensities was assessed along with the occurrence of El Niño events during 1990–2012. Cyclone intensities were represented by the minimum central pressure^24^, which was the lowest value of central pressure in each cyclone track. The strength of El Niño was evaluated by the Oceanic Niño index, which is used by National Oceanic and Atmospheric Administration (NOAA), USA, for identifying El Niño events in the tropical Pacific. The Oceanic Niño index is categorised into four classes: weak, moderate, strong and very strong.

We estimated the value of wetlands for cyclone protection from the relationship between TD/GDP and influential variables. This method is modified from Costanza, *et al*.^8^. The valuation of wetlands for cyclone protection consists of two avenues. To start with, regression analysis was conducted to examine the relationship between TD/GDP and wetland area, wind speed, the duration and minimum central pressure of cyclones consistently for both countries. In the analysis, cyclones with no significant wetlands in their tracks were excluded since our aim was to assess the role of wetlands rather than other ecosystems in attenuating cyclones. Moreover, too many zero values of wetland area will lever the regression and swamp the non-zero values, and thus making the regression less useful. Subsequently, the models derived from the above regression analysis were combined with data in terms of annual cyclone frequencies to estimate the annual value of wetlands for cyclone protection.

The effect of seawalls on the TD from cyclones in China cannot be directly evaluated since the geographic information of seawalls constructed in each year during 1990–2012 is not accessible. Therefore, the effect of seawalls accounts for part, if not all, of the variability in the relationship between TD/GDP and the independent variables but could not be accounted for due to the lack of geographic information. The seawalls were continuously being built during the study period but were incomplete before 2010. From 2010, seawalls were established along the China’s coastline^15^, and presumably effective, could account for the full strength of damage attenuation in cyclones. If seawalls can mitigate cyclones, the actual damage can reflect the effect, while the estimated damage definitely excludes the seawall influence which is not an explanatory variable in the regression model. By comparing the actual damage and estimated damage (from the regression analyses between TD/GDP and independent variables) after 2010, the effect of seawalls can be accounted for coarsely.

The collated data were combined to generate the values that are useful in our analysis. First of all, 100 × 100 km cyclone swaths were produced with the buffer function in geographic information systems, i.e. the average cyclone swath, and then overlaid on the wetland cover and GDP to produce GDP and wetland area in each cyclone swath. The total expected MV and the unit average annual MV were derived from the regression analysis from cyclones of a given GDP as well as the wetland area in swath. The total expected MV were calculated by multiplying the unit average annual MV and wetland area within 100 km of the coast. Subsequently, to compare with the result of Costanza, *et al*.^8^, data on storm frequency by state/territory and by pixel from historical cyclone tracks were collected. They are critical to deriving a surrogate for the annual probability of wetlands being hit by cyclones in specific categories (namely 1 to 5). Then a buffer zone of 100 km inland was created and 1 × 1 km pixels were generated^7^. Data on the frequencies of cyclones hitting each administrative region (state/territory/province/municipality) were collected and used to estimate the annual probability of the pixel being hit by cyclones of specific categories. These probabilities were used to estimate damage by pixel, along with GDP and area of wetlands in the pixel.

### Statistical and spatial analysis

Multiple regression analyses were conducted to evaluate the relationship between TD/GDP, wetland area, wind speed, and the duration and minimum central pressure of cyclones. Before regression analyses, data were checked for normality by the Shapiro-Wilk test (α = 0.05). Significant outliers were removed via Cook’s distance. Variables were log-transformed where data violate the normality assumption. Data were also checked for possible interactions among independent variables by the variance inflation factor^36^. Step-wise regression analyses were performed to select the best model for predicting TD/GDP using the independent variables. We used the AIC to test alternative models, and the model with the lowest AIC from an array of models was selected. Paired-sample t-test was performed to compare the actual and estimated TD/GDP in China after 2010. Mann-Whitney test was used to compare TD/GDP between Australia and China, due to the unbalanced numbers of samples. Functions of spatial analyses, such as buffer, aggregate/disaggregate, rasterize, extract and mask, were used to conduct spatial analysis.

All the analyses were conducted by R programming language^37^. The R packages ‘raster’^38^, ‘rasterVis’^39^, ‘rgdal’^40^, ‘sp’^41^, ‘rgeos’^42^, ‘colorspace’^43^, ‘maps’^44^ and ‘maptools’^45^ were used in the spatial analysis. The R package ‘rockchalk’^46^ was used to produce the 3D plot of multiple regression analyses. The R package ‘relaimpo’^47^ was used to calculate the variance explained by minimum central pressure and wetland area in the regression model of China. Part of the computing task was assisted by the third-party application of R in the 79-core high performance computer cluster Gowonda (Intel Xeon CPU X5650 processor@2.67 GHz, QDR 4 × InfiniBand Interconnect) under Linux environment.

### Data availability

Data are available upon request to oyxiaoguang@gmail.com.

## Electronic supplementary material

Supplementary material

## References

[1] Mitsch, W. J. & Gosselink, J. G. *Wetlands* (*4th Edition*). (John Wiley & Sons, Inc., 2007).

[2] Ouyang X, Guo F (2016). Paradigms of mangroves in treatment of anthropogenic wastewater pollution. Sci. Total Environ..

[3] Ouyang X, Lee SY (2014). Updated estimates of carbon accumulation rates in coastal marsh sediments. Biogeosciences.

[4] Nagelkerken I, Sheaves M, Baker R, Connolly RM (2015). The seascape nursery: a novel spatial approach to identify and manage nurseries for coastal marine fauna. Fish and Fisheries.

[5] Nemerow, N. L. In *Industrial Waste Treatment* 515–526 (Butterworth-Heinemann, 2007).

[6] UNEP. Marine and coastal ecosystems and human well-being: a synthesis report based on the findings of the Millennium Ecosystem Assessment. UNEP (2006).

[7] McGranahan G, Balk D, Anderson B (2007). The rising tide: assessing the risks of climate change and human settlements in low elevation coastal zones. Environ. Urban..

[8] Costanza R (2008). The value of coastal wetlands for hurricane protection. AMBIO: A Journal of the Human Environment.

[9] Spalding MD (2014). The role of ecosystems in coastal protection: Adapting to climate change and coastal hazards. Ocean Coast. Manag..

[10] Lee SY (2014). Ecological role and services of tropical mangrove ecosystems: a reassessment. Glob. Ecol. Biogeogr..

[11] Titus JG (2009). State and local governments plan for development of most land vulnerable to rising sea level along the US Atlantic coast. Environ. Res. Lett..

[12] Rosenzweig C (2011). Developing coastal adaptation to climate change in the New York City infrastructure-shed: process, approach, tools, and strategies. Clim. Chang..

[13] Brown S, Barton M, Nicholls R (2011). Coastal retreat and/or advance adjacent to defences in England and Wales. J. Coast. Conservat..

[14] Stancheva, M. *et al*. Expanding level of coastal armouring: case studies from different countries. *J. Coast. Res*. **1815** (2011).

[15] Ma Z (2014). Rethinking China’s new great wall. Science.

[16] Venkatachalam AJ, Price A, Chandrasekara S, Senaratna Sellamuttu S (2009). Risk factors in relation to human deaths and other tsunami (2004) impacts in Sri Lanka: the fishers’‐eye view. Aquatic Conservation: Marine and Freshwater Ecosystems.

[17] Chang SE (2006). Coastal ecosystems and tsunami protection after the December 2004 Indian Ocean tsunami. Earthquake Spectra.

[18] Das S, Vincent JR (2009). Mangroves protected villages and reduced death toll during Indian super cyclone. P. Natl. Acad. Sci. USA.

[19] Pinsky ML, Guannel G, Arkema KK (2013). Quantifying wave attenuation to inform coastal habitat conservation. Ecosphere.

[20] Wolanski, E. *Coastal protection in the aftermath of the Indian ocean tsunami: what role for forests and trees?* In: Braatz, S., Fortuna, S., Broadhead, J., Leslie, R. (Eds), Proceedings of an FAO Regional TechnicalWorkshop, Khao Lak, Thailand, 28–31 August 2006. FAO, Thailand, pp. 157–179 (2007).

[21] Nicholls N, Lavery B, Frederiksen C, Drosdowsky W, Torok S (1996). Recent apparent changes in relationships between the El Niño‐Southern Oscillation and Australian rainfall and temperature. Geophysical Research Letters.

[22] Hamilton SE, Casey D (2016). Creation of a high spatio‐temporal resolution global database of continuous mangrove forest cover for the 21st century (CGMFC‐21). Glob. Ecol. Biogeogr..

[23] Mcowen, C. J. *et al*. A global map of saltmarshes. *Biodiversity data journal* e11764, 10.3897/BDJ.5.e11764 (2017). 10.3897/BDJ.5.e11764 PMC5515097 28765720

[24] Mendelsohn R, Emanuel K, Chonabayashi S, Bakkensen L (2012). The impact of climate change on global tropical cyclone damage. Nat. Clim. Chang..

[25] Barbier EB (2016). The protective service of mangrove ecosystems: A review of valuation methods. Mar. Pollut. Bull..

[26] Webster PJ, Holland GJ, Curry JA, Chang H-R (2005). Changes in tropical cyclone number, duration, and intensity in a warming environment. Science.

[27] Gedan KB, Kirwan ML, Wolanski E, Barbier EB, Silliman BR (2011). The present and future role of coastal wetland vegetation in protecting shorelines: answering recent challenges to the paradigm. Clim. Chang..

[28] Barbier EB (2012). A spatial model of coastal ecosystem services. Ecol Econ.

[29] Barbier EB (2007). Valuing ecosystem services as productive inputs. Econ. Policy.

[30] Trenberth K (2005). Uncertainty in hurricanes and global warming. Science.

[31] Barbier EB (2011). The value of estuarine and coastal ecosystem services. Ecol. Monogr..

[32] McIvor, A., Möller, I., Spencer, T. & Spalding, M. Reduction of wind and swell waves by mangroves In: Natural Coastal Protection Series: Report 1. The Nature Conservancy, University of Cambridge, and Wetlands International, Cambridge, UK (2012).

[33] Koch EW (2009). Non‐linearity in ecosystem services: temporal and spatial variability in coastal protection. Front. Ecol. Environ..

[34] Tanaka N, Sasaki Y, Mowjood M, Jinadasa K, Homchuen S (2007). Coastal vegetation structures and their functions in tsunami protection: experience of the recent Indian Ocean tsunami. Landsc. Ecol. Eng..

[35] Yap W (2015). A historical typhoon database for the southern and eastern Chinese coastal regions, 1951 to 2012. Ocean Coast. Manag..

[36] Zuur, A. F. *et al*. *Statistics for biology and health* (Springer, 2009).

[37] R: A language and environment for statistical computing. R Foundation for Statistical Computing. Vienna, Austria (2014).

[38] Hijmans, R. J. raster: Geographic Data Analysis and Modeling. R package version 2.5–8. https://CRAN.R-project.org/package=raster (2016).

[39] Lamigueiro, O. P. & Hijmans, R. meteo Forecast. R package version 0.40 (2016).

[40] Bivand, R., Keitt, T. & Rowlingson, B. rgdal: Bindings for the Geospatial Data Abstraction Library. R package version 1.1–10. https://CRAN.R-project.org/package=rgdal (2016).

[41] Pebesma, E. J. & Bivand, R. S. Classes and methods for spatial data in R. R News **5**(2), http://cran.r-project.org/doc/Rnews/ (2005).

[42] Bivand, R. & Rundel, C. rgeos: Interface to Geometry Engine - Open Source (GEOS). R package version 0.3–19. https://CRAN.R-project.org/package=rgeos (2016).

[43] Ihaka, R., Murrell, P., Hornik, K., Fisher, J. C. & Zeileis, A. colorspace: Color Space Manipulation. R package version 1.2–6. URL http://CRAN.R-project.org/package=colorspace (2015).

[44] Minka, T. P. & Deckmyn, A. maps: Draw Geographical Maps. R package version 3.1.0. https://CRAN.R-project.org/package=maps (2016).

[45] Bivand, R. & Lewin-Koh, N. maptools: Tools for Reading and Handling Spatial Objects. R package version 0.8-39. https://CRAN.R-project.org/package=maptools (2016).

[46] Johnson, P. E. rockchalk: Regression Estimation and Presentation. R package version 1.8.101. https://CRAN.R-project.org/package=rockchalk (2016).

[47] Grömping U (2006). Relative importance for linear regression in R: the package relaimpo. J. Stat. Softw..

[48] Ko J-Y, Day JW, Lane RR, Day JN (2004). A comparative evaluation of money-based and energy-based cost–benefit analyses of tertiary municipal wastewater treatment using forested wetlands vs. sand filtration in Louisiana. Ecol. Econ.

[49] Ghermandi A, Fichtman E (2015). Cultural ecosystem services of multifunctional constructed treatment wetlands and waste stabilization ponds: Time to enter the mainstream?. Ecol. Eng..

[50] Woodward RT, Wui Y-S (2001). The economic value of wetland services: a meta-analysis. Ecol. Econ.

[51] Camacho-Valdez V, Ruiz-Luna A, Ghermandi A, Nunes PALD (2013). Valuation of ecosystem services provided by coastal wetlands in northwest Mexico. Ocean Coast. Manag..

[52] Mukherjee N (2014). Ecosystem service valuations of mangrove ecosystems to inform decision making and future valuation exercises. PloS one.

