# Supplementary material for: Spatially-explicit valuation of coastal wetlands for cyclone mitigation in Australia and China
Source: Sci Rep. 2018 Feb 14;8:3035. doi: 10.1038/s41598-018-21217-z (PMC5813045; doi:10.1038/s41598-018-21217-z)
Supplement: Supplementary file 1 — Supplementary material [file 41598_2018_21217_MOESM1_ESM.doc]

**Supplementary materials for**

**Spatially-explicit valuation of coastal wetlands for cyclone mitigation**

**in Australia and China**

Xiaoguang Ouyang, Shing Yip Lee, Rod M. Connolly, Martin Kainz

**This file includes**

Supplementary Text

Table S1

Table S2

**Supplementary Text**

The linear relationship between ln(TP/GDP) and wetland area in cyclone swath in Australia is presented as below


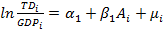
 (1)

In equation (1),
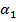
 and
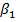
 are regression parameters,
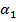
 = -1.044 and
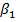
 = -3.812×10^-5^, respectively; TD_i_ is total economic damage from cyclone i (in constant 2011 USD); GDP_i_ is GDP in the swath of cyclone i; A_i_ is area of wetland in the swath of cyclone i; μ_i_ is residual standard error.

The relationship between ln(TP/GDP) and ln (minimum pressure), wetland area in cyclone swath in China is presented as below


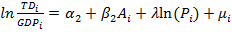
 (2)

In equation (2),
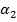
,
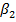
 and
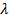
are regression parameters,
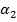
, =-123.7,
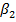
 =-1.475×10^-3^ and
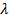
= -18.52, respectively; P_i_ is the minimum central pressure from cyclone i.

Based on equation (1) and (2), the total economic damages for cyclone i in Australia and China are estimated as below


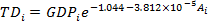
(3)


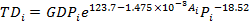
(4)

The regression models show that TP/GDP decreases with increasing wetland area. The result is in accordance with that of Costanza, et al^. 1^, although the regression models derived in both studies are different. Our study further underpins the viewpoint that wetlands, especially coastal wetlands, are significant to attenuating the impact of cyclones ^2,3^.

The differences in TD_i_ with a loss of wetland area of
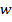
, i.e. the ‘avoided damage’ per unit area of wetlands in Australia and China, are expressed as below


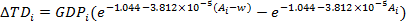
(5)


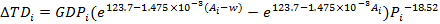
 (6)

Presumably
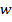
 is small in relation to A in equations (5) and (6), this represents the estimated ‘marginal value’ (MV) per unit area of wetlands in preventing storm damage from a specific cyclone.

The annual probability of coastal regions being struck by cyclones of various categories was estimated, to assess the annual value of wetlands for cyclone protection. Data for each state/territory in Australia that have been struck since 1990 are utilized to estimate the historical frequency of cyclone occurrence. The annual expected MV for an average cyclone swath in each state/territory in Australia is calculated as below


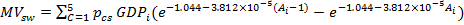
 (7)

The annual expected MV for an average cyclone swath in each province/municipality in China is calculated as below


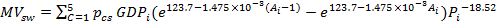
 (8)

In equations (7) and (8), s is state (or province, municipality); sw is average swath in s; p_cs_ is the probability of a cyclone of category c hitting s in a given year. Table S2 lists MV_i_ for each of the cyclones for unit areas of one ha in both Australia and China.

Table S1 Parameter estimates of the regression relationship between TD/GDP and wetland area and/or minimum central pressure of cyclones.

a) Australia

| Parameter | Coefficient | Upper 95% CI | Lower 95% CI | SE | t | P |
| --- | --- | --- | --- | --- | --- | --- |
| α | -1.044 | -0.098 | -1.998 | 0.410 | -2.550 | <0.05 |
| β | -3.812×10^-5^ | -1.221×10^-5^ | -6.402×10^-5^ | 1.123×10^-5^ | -3.393 | <0.01 |

b) China

| Parameter | Coefficient | Upper 95% CI | Lower 95% CI | SE | t | P |
| --- | --- | --- | --- | --- | --- | --- |
| α | 123.700 | 243.108 | 4.361 | 60.11 | 2.058 | <0.05 |
| β | -1.475×10^-3^ | -4.859×10^-5^ | -0.003 | 7.185×10^-4^ | -2.053 | <0.05 |
| λ | -18.520 | -1.149 | -35.900 | 8.750 | -2.117 | <0.05 |

Table S2 Economic damages, GDP and related indicators of cyclones in the oceans around Australia and China during 1990-2012

| **Name** | **Year** | **Observed TD**  **(million $)*** | **GDP in cyclone swath (million $)*** | **Wetland area in cyclone swath (ha)** | **Duration of cyclones (hour)** | **Minimum central**  **pressure (hPa)** | **Maximum sustained**  **wind speed (m/s)** | **Estimated MV**  **($ ha^-1^)*** | **TD/GDP** | **Estimated TD/GDP** |
| --- | --- | --- | --- | --- | --- | --- | --- | --- | --- | --- |
| China | | | | | | | | | | |
| Percy | 1990 | 89.95 | 11286.94 | 116.94 | 210 | 950 | 148 | 526704.1 | 0.00797 | 0.031614 |
| Tasha | 1990 | 1031.78 | 3143.00 | 340.38 | 126 | 970 | 101.75 | 71718.03 | 0.32828 | 0.015459 |
| Mike | 1990 | 1248.09 | 1494.50 | 114.85 | 240 | 915 | 277.8 | 140199.4 | 0.835125 | 0.063553 |
| Abe | 1990 | 1734.09 | 23407.13 | 316.31 | 180 | 945 | 138.75 | 897568.4 | 0.074084 | 0.025978 |
| Zeke | 1991 | 301.07 | 1927.18 | 344.93 | 90 | 970 | 120.25 | 43680.97 | 0.156223 | 0.015355 |
| Brendan | 1991 | 306.82 | 2463.50 | 571.54 | 78 | 980 | 111 | 33057.41 | 0.124546 | 0.009091 |
| Fred | 1991 | 361.33 | 2923.25 | 233.94 | 120 | 960 | 138.75 | 94554.78 | 0.123605 | 0.021913 |
| Eli | 1992 | 654.47 | 2581.41 | 344.93 | 96 | 965 | 130 | 64386.32 | 0.253532 | 0.016898 |
| Mark | 1992 | 28.96 | 4650.80 | 341.03 | 78 | 990 | 85 | 72651.38 | 0.006228 | 0.010583 |
| Chuck | 1992 | 144.80 | 3177.43 | 230.08 | 120 | 965 | 129.5 | 93882.14 | 0.045572 | 0.020017 |
| Gary | 1992 | 262.87 | 2313.92 | 228.60 | 60 | 980 | 101.75 | 51492.34 | 0.113604 | 0.015076 |
| Ted | 1992 | 1373.76 | 43898.39 | 316.31 | 66 | 990 | 92.5 | 711215.4 | 0.031294 | 0.010976 |
| Koryn | 1993 | 531.40 | 3851.78 | 572.68 | 168 | 905 | 194.25 | 225435.6 | 0.137963 | 0.039651 |
| Tasha | 1993 | 1051.77 | 3098.17 | 114.14 | 90 | 980 | 120.25 | 81624.61 | 0.33948 | 0.017849 |
| Abe | 1993 | 30.49 | 3884.32 | 456.82 | 108 | 955 | 157.25 | 99623.47 | 0.007849 | 0.017375 |
| Becky | 1993 | 1108.96 | 3749.44 | 457.72 | 60 | 980 | 101.75 | 59510.24 | 0.295768 | 0.010753 |
| Dot | 1993 | 706.90 | 5610.25 | 227.54 | 60 | 965 | 129.5 | 166385.5 | 0.126002 | 0.020092 |
| Luke | 1994 | 23.43 | 2008.29 | 116.04 | 126 | 990 | 85 | 43718.94 | 0.011666 | 0.014748 |
| Sharons | 1994 | 12.01 | 1793.74 | 113.32 | 36 | 996 | 75 | 35054.5 | 0.006698 | 0.013240 |
| Harry | 1994 | 113.14 | 4581.50 | 344.93 | 78 | 990 | 85 | 71158.39 | 0.024695 | 0.010522 |
| Russ | 1994 | 1549.57 | 2802.52 | 340.70 | 102 | 985 | 92.5 | 48106.21 | 0.552921 | 0.011629 |
| Fred | 1994 | 4080.23 | 5290.56 | 106.96 | 174 | 925 | 185 | 410565.5 | 0.771229 | 0.052574 |
| Janis | 1995 | 755.20 | 67543.45 | 316.21 | 102 | 990 | 85 | 1094468 | 0.011181 | 0.010978 |
| Sibyl | 1995 | 67.85 | 2779.30 | 113.32 | 126 | 985 | 95 | 66718.18 | 0.024414 | 0.016263 |
| Helen | 1995 | 627.35 | 11536.70 | 340.79 | 84 | 985 | 92.5 | 198005.9 | 0.054378 | 0.011627 |
| Kent | 1995 | 796.97 | 31318.41 | 680.67 | 138 | 945 | 157.25 | 701637.8 | 0.025447 | 0.015178 |
| Lois | 1995 | 857.61 | 8310.97 | 233.80 | 102 | 980 | 92.5 | 183532.7 | 0.10319 | 0.014961 |
| Ted | 1995 | 237.31 | 5063.30 | 796.85 | 129 | 985 | 92.5 | 44349.37 | 0.046869 | 0.005934 |
| Frankie | 1996 | 331.03 | 6033.12 | 115.08 | 66 | 975 | 120 | 174501.4 | 0.054869 | 0.019595 |
| Herb | 1996 | 14623.45 | 20637.66 | 213.70 | 222 | 925 | 175.75 | 1368245 | 0.708581 | 0.044915 |
| Sally | 1996 | 3751.27 | 8533.36 | 228.60 | 96 | 940 | 157.25 | 410859.1 | 0.439601 | 0.032618 |
| Victor | 1997 | 728.74 | 36566.40 | 450.01 | 75 | 980 | 111 | 587007.8 | 0.019929 | 0.010876 |
| Zita | 1997 | 796.70 | 9821.69 | 344.93 | 54 | 980 | 101.75 | 184103.4 | 0.081116 | 0.012699 |
| Amber | 1997 | 126.16 | 18436.61 | 106.96 | 189 | 950 | 148 | 873103.4 | 0.006843 | 0.032083 |
| Maggie | 1999 | 386.30 | 44117.24 | 796.06 | 132 | 955 | 138.75 | 686032.8 | 0.008756 | 0.010535 |
| Sam | 1999 | 198.70 | 55988.07 | 458.39 | 90 | 980 | 101.75 | 887749.8 | 0.003549 | 0.010742 |
| Wendy | 1999 | 2143.77 | 6969.55 | 343.75 | 12 | 996 | 64.75 | 96957.62 | 0.307591 | 0.009425 |
| York | 1999 | 178.85 | 39723.95 | 454.77 | 84 | 980 | 101.75 | 633233.4 | 0.004502 | 0.010799 |
| Dan | 1999 | 2281.54 | 21361.57 | 116.94 | 150 | 955 | 148 | 904486.7 | 0.106806 | 0.028685 |
| Jelawat | 2000 | 48.26 | 30607.34 | 105.55 | 222 | 940 | 157.25 | 1766953 | 0.001577 | 0.039110 |
| Maria | 2000 | 269.78 | 17665.44 | 226.72 | 90 | 985 | 74 | 358747.5 | 0.015271 | 0.013758 |
| Wukong | 2000 | 294.65 | 5284.59 | 117.00 | 108 | 955 | 138.75 | 223736.7 | 0.055756 | 0.028682 |
| Durian | 2001 | 675.20 | 7832.73 | 228.60 | 60 | 970 | 110 | 210766.4 | 0.086202 | 0.018230 |
| Utor | 2001 | 4392.00 | 64355.30 | 684.62 | 108 | 960 | 111 | 1070790 | 0.068246 | 0.011272 |
| Yutu | 2001 | 136.00 | 11290.34 | 338.27 | 54 | 975 | 101.75 | 234959 | 0.012046 | 0.014098 |
| Fitow | 2001 | 340.80 | 14978.76 | 342.17 | 12 | 990 | 64.75 | 233593.6 | 0.022752 | 0.010565 |
| Nari | 2001 | 1280.00 | 108291.45 | 908.31 | 282 | 960 | 138.75 | 1295453 | 0.01182 | 0.008104 |
| Mekkhala | 2002 | 163.84 | 14484.86 | 343.22 | 72 | 990 | 85 | 225542.8 | 0.011311 | 0.010549 |
| Hagupit | 2002 | 51.70 | 9708.44 | 1024.50 | 24 | 990 | 85 | 55340.98 | 0.005325 | 0.003862 |
| Vongfong | 2002 | 142.04 | 6802.31 | 116.04 | 45 | 985 | 100 | 162639.2 | 0.020882 | 0.016198 |
| Sinlaku | 2002 | 159.14 | 14314.07 | 109.51 | 234 | 950 | 148 | 675326.2 | 0.011118 | 0.031962 |
| Krovanh | 2003 | 392.11 | 15861.25 | 460.43 | 138 | 970 | 120 | 303194.6 | 0.024722 | 0.012950 |
| Morakot | 2003 | 48.05 | 41696.98 | 111.49 | 54 | 992 | 85 | 880298.8 | 0.001152 | 0.014303 |
| Koni | 2003 | 25.26 | 9306.42 | 115.75 | 108 | 975 | 110 | 268911.9 | 0.002715 | 0.019576 |
| Imbudo | 2003 | 154.99 | 16527.97 | 909.87 | 186 | 935 | 166.5 | 321571.9 | 0.009377 | 0.013181 |
| Dujuan | 2003 | 373.52 | 76821.03 | 2503.07 | 102 | 950 | 148 | 106157.9 | 0.004862 | 0.000936 |
| Nepartak | 2003 | 304.39 | 10973.06 | 226.14 | 156 | 970 | 120.25 | 296342.9 | 0.02774 | 0.018296 |
| Aere | 2004 | 14.49 | 42211.00 | 111.49 | 150 | 955 | 148 | 1801710 | 0.000343 | 0.028917 |
| Haitang | 2005 | 1535.67 | 28058.32 | 110.75 | 174 | 920 | 194.25 | 2393896 | 0.054731 | 0.057800 |
| Matsa | 2005 | 1188.05 | 53449.69 | 105.88 | 168 | 950 | 148 | 2535247 | 0.022227 | 0.032134 |
| Talim | 2005 | 2705.57 | 31578.61 | 111.49 | 72 | 985 | 175.75 | 760108.7 | 0.085677 | 0.016307 |
| Khanun | 2005 | 2443.11 | 50268.51 | 105.88 | 144 | 945 | 157.25 | 2629149 | 0.048601 | 0.035433 |
| Longwang | 2005 | 349.02 | 59377.80 | 111.49 | 168 | 930 | 175.75 | 4142301 | 0.005878 | 0.047261 |
| Chanchu | 2006 | 638.02 | 87085.90 | 219.50 | 228 | 930 | 175.75 | 5180551 | 0.007326 | 0.040301 |
| Bilis | 2006 | 4466.17 | 40173.25 | 109.99 | 144 | 970 | 111 | 1287672 | 0.111173 | 0.021715 |
| Prapiroon | 2006 | 1208.89 | 17346.55 | 340.96 | 90 | 970 | 120.25 | 395481.3 | 0.06969 | 0.015445 |
| Saomai | 2006 | 3371.46 | 21554.66 | 1073.54 | 132 | 925 | 194.25 | 402015.3 | 0.156414 | 0.012635 |
| Neuguri | 2008 | 56.59 | 25930.00 | 796.45 | 108 | 960 | 148 | 365832.8 | 0.002183 | 0.009558 |
| Fengshen | 2008 | 202.12 | 26719.60 | 568.09 | 150 | 945 | 166.5 | 706742.1 | 0.007565 | 0.017919 |
| Kalmaegi | 2008 | 18.48 | 81233.09 | 422.19 | 84 | 970 | 120.25 | 1642899 | 0.000227 | 0.013701 |
| fung-Wong | 2008 | 95.86 | 62948.88 | 445.82 | 102 | 960 | 138.75 | 1489640 | 0.001523 | 0.016032 |
| Kammuri | 2008 | 92.40 | 56901.42 | 914.41 | 66 | 975 | 92.5 | 506225.3 | 0.001624 | 0.006027 |
| Higos | 2008 | 7.51 | 33114.70 | 1482.61 | 30 | 996 | 65 | 85874.34 | 0.000227 | 0.001757 |
| Nuri | 2008 | 67.43 | 48419.82 | 912.09 | 126 | 955 | 138.75 | 634498.3 | 0.001393 | 0.008878 |
| Hagupit | 2008 | 951.71 | 72018.09 | 572.53 | 132 | 935 | 166.5 | 2304617 | 0.013215 | 0.021679 |
| Linfa | 2009 | 110.94 | 42139.76 | 222.70 | 102 | 975 | 111 | 1039934 | 0.002633 | 0.016719 |
| Nangka | 2009 | 6.40 | 22028.11 | 454.86 | 84 | 994 | 74 | 269988.6 | 0.000291 | 0.008303 |
| Molave | 2009 | 109.04 | 44695.01 | 909.22 | 72 | 975 | 120.25 | 400682.8 | 0.00244 | 0.006073 |
| Goni | 2009 | 295.96 | 44795.41 | 456.80 | 90 | 990 | 74 | 589912.3 | 0.006607 | 0.008922 |
| Soudelor | 2009 | 6.48 | 60036.48 | 460.01 | 24 | 992 | 65 | 758025.1 | 0.000108 | 0.008554 |
| Mujigae | 2009 | 6.92 | 36834.83 | 116.04 | 48 | 994 | 74 | 744167.2 | 0.000188 | 0.013687 |
| Koppu | 2009 | 392.89 | 47613.58 | 342.50 | 42 | 975 | 120.25 | 984697.1 | 0.008252 | 0.014011 |
| Parma | 2009 | 79.44 | 49465.03 | 403.18 | 330 | 930 | 185 | 2244209 | 0.001606 | 0.030736 |
| Conson | 2010 | 79.82 | 51266.01 | 574.70 | 144 | 970 | 129.5 | 827964.8 | 0.001557 | 0.010941 |
| Chanthu | 2010 | 308.67 | 51266.01 | 684.98 | 90 | 965 | 129.5 | 774351 | 0.006021 | 0.010233 |
| Lionrock | 2010 | 3.48 | 59492.83 | 225.28 | 108 | 985 | 92.5 | 1210746 | 5.85E-05 | 0.013787 |
| Fanapi | 2010 | 550.69 | 80793.37 | 338.27 | 126 | 935 | 175.75 | 3652543 | 0.006816 | 0.030627 |
| Sarika | 2011 | 248.00 | 16420.38 | 338.08 | 42 | 996 | 75 | 230349.9 | 0.015103 | 0.009504 |
| Nock-Ten | 2011 | 377.00 | 53584.56 | 463.79 | 120 | 985 | 92.5 | 767088.5 | 0.007036 | 0.009698 |
| Nanmadol | 2011 | 83.30 | 21396.83 | 116.94 | 180 | 925 | 185 | 1636198 | 0.003893 | 0.051805 |
| Nesat | 2011 | 421.50 | 8170.06 | 921.74 | 162 | 950 | 148 | 116325.2 | 0.05159 | 0.009646 |
| Talim | 2012 | 230.09 | 22565.86 | 116.04 | 150 | 925 | 92.5 | 1727884 | 0.010196 | 0.051874 |
| Vicente | 2012 | 321.32 | 40300.92 | 226.78 | 78 | 950 | 148 | 1599353 | 0.007973 | 0.026885 |
| Saola | 2012 | 625.44 | 26859.73 | 109.99 | 150 | 960 | 129.5 | 1043083 | 0.023285 | 0.026309 |
| Haikui | 2012 | 1464.99 | 33894.31 | 423.84 | 162 | 965 | 120.25 | 752517.4 | 0.043222 | 0.015041 |
| Kai-tak | 2012 | 255.89 | 52604.17 | 1036.24 | 126 | 970 | 120.25 | 430076.5 | 0.004864 | 0.005539 |
| Son-Tinh | 2012 | 192.40 | 14069.80 | 460.16 | 138 | 945 | 157.25 | 436372.3 | 0.013675 | 0.021011 |
| Australia | | | | | | | | | | |
| Carlos | 2011 | 16.20 | 45228.47 | 144536 | 312 | 969 | 33.33 | 2456.59 | 0.000358 | 0.001425 |
| Ellie | 2009 | 75.78 | 2846.55 | 23590.86 | 36 | 988 | 20.83 | 13688.65 | 0.030227 | 0.143233 |
| Frank | 1995 | 25.10 | 20023.03 | 11878.65 | 171 | 950 | 43.06 | 98013.2 | 0.002185 | 0.223842 |
| George | 2007 | 18.48 | 38130.94 | 60295.71 | 318 | 902 | 56.94 | 48162.84 | 0.000517 | 0.035349 |
| Glenda | 2006 | 1191.42 | 1363.40 | 0 | 222 | 910 | 56.94 | 17783.22 | 0.899109 | 0.352044 |
| Joy | 1990 | 116.55 | 3189.66 | 23362.48 | 217 | 940 | 45.83 | 31995.18 | 0.020064 | 0.144485 |
| Larry | 2006 | 1358.09 | 25946.62 | 11808.69 | 150 | 940 | 56.94 | 215760.3 | 0.053854 | 0.22444 |
| Monica | 2006 | 6.30 | 147158.30 | 72410.3 | 282 | 916 | 69.44 | 121449.9 | 0.000044 | 0.022275 |
| Steve | 2000 | 92.84 | 49995.19 | 11878.65 | 334 | 975 | 41.67 | 275175.1 | 0.002879 | 0.223842 |
| Ului | 2010 | 76.43 | 63378.30 | 23296.78 | 148 | 937 | 59.72 | 56797.04 | 0.00743 | 0.144848 |
| Vance | 1999 | 158.20 | 95840.80 | 48429.04 | 226 | 910 | 59.72 | 25084.31 | 0.01336 | 0.05557 |
| Helen | 2008 | 36.73 | 9541.47 | 0 | 222 | 975 | 26.39 | 108488.3 | 0.004544 | 0.352044 |
| Ingrid | 2005 | 35.63 | 3048.009 | 0 | 309 | 924 | 63.89 | 33139.32 | 0.014429 | 0.352044 |
| Inigo | 2003 | 20.48 | 8404.079 | 0 | 201 | 900 | 66.67 | 73022.83 | 0.003764 | 0.352044 |
| Winsome | 2001 | 2.149 | 6593.711 | 0 | 120 | 981 | 33.06 | 101597.1 | 0.000284 | 0.352044 |
| Olivia | 1996 | 200.89 | 18465.04 | 0 | 147 | 925 | 54.17 | 171241.5 | 0.015744 | 0.352044 |
| Lua | 2012 | 99.44 | 28070.87 | 0 | 183 | 930 | 45.83 | 123642.8 | 0.010793 | 0.352044 |

* Values are expressed as 2011 constant USD. TD refers to total damage

**References**

1 Costanza, R. *et al.* The value of coastal wetlands for hurricane protection. *AMBIO: A Journal of the Human Environment* **37**, 241-248 (2008).

2 Gedan, K. B., Kirwan, M. L., Wolanski, E., Barbier, E. B. & Silliman, B. R. The present and future role of coastal wetland vegetation in protecting shorelines: answering recent challenges to the paradigm. *Clim. Chang.* **106**, 7-29 (2011).

3 Barbier, E. B. A spatial model of coastal ecosystem services. *Ecol. Econ,* **78**, 70-79 (2012).
